# Creation of a linked cohort of children and their parents in a large, national electronic health record dataset

**DOI:** 10.1097/MD.0000000000026950

**Published:** 2021-08-13

**Authors:** Heather Angier, Sophia Giebultowicz, Jorge Kaufmann, John Heintzman, Jean O’Malley, Laura Moreno, Jennifer E. DeVoe

**Affiliations:** a Oregon Health & Science University; b OCHIN, Inc., Portland, OR.

**Keywords:** child health, family health, health services research

## Abstract

To examine which parental health care and health factors are most strongly associated with a child's receipt of recommended care we must be able to link children to their parents in electronic health record data. Yet, there is not an easy way to link these data.

To identify a national cohort of children that link to at least one parent in the same electronic health record dataset and describe their demographics.

Methodology to link parents and children in electronic health records and descriptive sociodemographic data.

Children with at least one encounter with a primary care clinician between Januray 1, 2007 and December 12, 2018 to a community health center in the OCHIN national network. We identified parents of these children who also had at least one encounter to a community health center in the network using emergency contact and guarantor record fields.

A total of 227,552 children had parents with a linkable patient record. After exclusions, our final cohort included 213,513 distinct children with either one or two parent-links. 82% of children linked to a mother only, 14% linked to a father only, and 4% linked to both a mother and a father. Most families consisted of only one linked child (61%).

We were able to link 33% of children to a parent in electronic health record data from a large network of community health centers across the United States. Further analyses utilizing these linkages will allow examination of the multi-level factors that impact a child's receipt of recommended health care.

## 1 Introduction

Parental health insurance and health status are associated with their children's health insurance and receipt of health care.^[1–7]^ For example, mothers with less than excellent health have increased odds of having a child with less than excellent health.^[5]^ Maternal depression (compared to no depression) is associated with lower rates of well-child checks and recommended immunizations, and poor child health outcomes.^[6]^ Paternal depression is associated with reports of psychological distress in children at multiple ages.^[8]^ Additionally, adolescent daughters of mothers with a routine doctor visit in the previous 12 months are one and a half times more likely to also have a routine doctor visit in the last 12 months.^[9]^ There is a causal link between parent and child health insurance coverage,^[10,11]^ and a strong association between parental coverage and timely receipt of health care for children. For example, children of Medicaid-enrolled parents experienced a 29% increased probability of receiving an annual well-child visit.^[12]^

**Figure 1:**
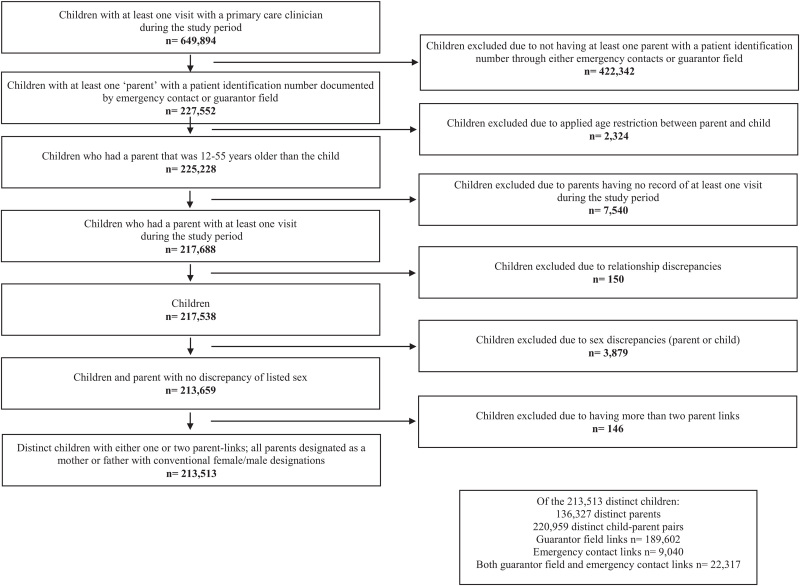
Consort diagram to define children and linked parent(s) using a national network of electronic health record data.

Many of these previous studies used cross-sectional survey data to answer questions about the health care services received by parents and children. Electronic health record (EHR) data provide a unique opportunity to assess longitudinal connections between parents’ and children's health care and health outcomes without self-report biases, especially for tracking longitudinal utilization, receipt of recommended care, and health status over time. EHR data also have great potential to facilitate a better understanding of multi-level influences (ie, individual, parent, family, and community-level) on children's receipt of recommended health care. Yet, linking children to their parents within EHR data is challenging. As most EHR datasets do not create direct linkages for family members, there is a need to develop methodologies to do so. Here, we applied a previously validated algorithm,^[13]^ which utilized EHR data to link children to parents in Oregon, to a large multi-state network of community health center (CHC) patients that has a centralized instance of the Epic© EHR hosted and maintained by a non-profit health information technology organization. CHCs function as the nation's health care safety net, providing health care to adults and children regardless of health insurance status.^[14]^ We describe how we applied the previous algorithm to identify a national cohort of children that link to at least one parent in the same clinical dataset and present the demographics of the cohort (Fig. 1).

## 2 Methods

We used EHR data from OCHIN's national network of CHCs serving >2 million medically underserved patients from 18 states to link parents and children.^[15,16]^ The patients seen in these CHCs are representative of patients seen in CHCs nationally.^[14]^ All included clinics provide comprehensive, coordinated primary care to patients of all ages. Each patient has only 1 patient identification (ID) number. After linking parent and child, we used sociodemographic data from the Accelerating Data Value Across a National Community Health Center (ADVANCE) clinical research network (CRN), a member of PCORnet. OCHIN leads the ADVANCE CRN and selected EHR data is contained within its data warehouse.

We applied our previously validated algorithm to all OCHIN community network CHCs.^[13]^ We included information from all children (aged 0–17) with at least 1 encounter with a primary care clinician from January 1, 2007 to December 12, 2018 (n = 649,894). We then used emergency contact information and guarantor records to identify children with parents who also had a patient record (n = 227,552). Parents also had to have at least one encounter at a networked CHC, which would give them a patient ID within the same linked CHC dataset for inclusion in the linkages.

For emergency contact we used an already-populated data point that specified whom the clinic should call if there was an urgent need. If the emergency contact was also a patient within the network, they had a patient ID. Attached to these contacts are relationship designations that include “mother” and “father," among others. There are five different emergency contact fields: mother, father, emergency contact 1, emergency contact 2, and guardian. If emergency contact 1, emergency contact 2, or guardian were populated, they contained a description of the relationship. We included children in our cohort if their emergency contact was someone with a patient ID that was designated as a mother or a father. The emergency contact field does not allow duplicate mothers to be listed. We identified 9040 children that could be linked to a parent in the emergency contact field alone.

In addition to emergency contact records we utilized guarantor records, which specify the individual responsible for payment (and a patient ID if they are also a patient within the network). These records also indicate the relationship of the guarantor to the patient. Many relationships are contained in the guarantor records, for example, aunt, brother, daughter, employer, grandmother, spouse, among others. The guarantor contact records can change over time and allow listing of up to four potential mothers and two potential fathers. To be included in our cohort, the guarantor record had to specify mother or father as a relationship; we excluded all other relationships. If a mother or father was specified as anything besides a mother or father in the additional guarantor fields, they were also excluded from our cohort. We identified 189,602 children that could be linked to a parent in the guarantor field alone. Some links were found in both the emergency contact and guarantor field (n = 22,317); none were discrepant.

From the linked emergency contacts and guarantor records we narrowed the children down to those who had parents who were 12 to 55 years older than the child (n = 225,228) in an attempt to prevent siblings from being captured as parents (per our previous algorithm).^[13]^ We kept only children with parents who had a record of at least one encounter during the same time period listed above for the children (n = 217,688). At this point, we excluded 150 children with discrepancies in their EHR record ID (ie, they had >1 ID number). We also excluded another 3879 children who had themselves or whose parents had a discrepancy in sex, meaning the child was listed as both male and female in their records or the child was linked to a mother that was listed as male in the parent medical record, or vice versa. Lastly, we excluded 146 children because they were linked to >2 parents.

We performed descriptive statistics with sociodemographic data from the EHR, including sex, age at first encounter, preferred language, region of the country, payer and federal poverty level at first encounter (or first encounter where these data were available), number of parental linkages, time in the study, average number of encounters per year, chronic conditions listed on the problem list, and age at death, if applicable. We also describe several characteristics of all children.

As the links created thus far were from one child to one parent only, we created family units to understand how many children were linked to 2 parents versus a mother or a father only. To create these categories, we took the full list of distinct children in the final cohort and joined the child's patient ID to the parent(s) patient ID. By doing this we were able to determine the children that linked to a mother only, a father only, a mother and a father, two mothers, or two fathers. Here, parents may be linked to more than one distinct family unit.

This study was reviewed and approved by the Oregon Health & Science University Institutional Review Board (STUDY00019958) with a waiver of consent and authorization, as the research involves minimal risk, does not adversely affect the rights of subjects, and could not be practicably carried out without the waiver.

## 3 Results

Our final cohort included 213,513 distinct children with either one or two parent-links; all parents were designated as a mother or a father with conventional female/male designations. Linked to these distinct children, were 126,327 parents and 220,959 child-parent pairs. 86% of links derived from the guarantor list (n = 189,602), 4% from emergency contacts (n = 9040), and 10% were found in both the guarantor list and emergency contacts (n = 22,317). (See Figure 1).

Half the children were female (50%), 44% reported Hispanic ethnicity, 27% reported white race, and 36% preferred a non-English language. Most children (97%) linked to one parent and had between 1 and 3 encounters per year (52%). Seventy-seven percent of children had Medicaid recorded at their first encounter (or the first encounter where these data were available). The majority of children did not have any chronic conditions (63%).

Of the parents, 114,395 were mothers and 21,932 were fathers. There were 42% of linked mothers with Hispanic ethnicity and 28% of linked fathers. 57% of mothers had Medicaid and 11% had private coverage, whereas 42% of fathers had Medicaid and 21% had private coverage recorded at their first encounter (or the first encounter where these data were available). 35% of mothers and 56% of fathers had between 1 and 3 encounters per year (see Table 1).

**Table 1:** Characteristics of the study sample, 2007–2018.

	Group, No. (%)		
Characteristic	Children	Mothers	Fathers
	N = 213,513	N = 114,395	N = 21,932
Female	106,372 (49.8%)	114,395 (100.0%)	0 (0.0%)
Age at first encounter^∗^	Median = 5	Median = 31	Median = 39
Race/ethnicity			
*Non-Hispanic white*	58,468 (27.4%)	36,445 (31.9%)	8163 (37.2%)
*Non-Hispanic Black*	30,135 (14.1%)	16,867 (14.7%)	2109 (9.6%)
*Hispanic*	93,020 (43.6%)	47,822 (41.8%)	6076 (27.7%)
*Other*	31,890 (14.9%)	13,261 (11.6%)	5584 (25.5%)
English Language Preferred	136,083 (63.7%)	71,810 (62.8%)	12,569 (57.3%)
Region^†^			
*Northeast*	20,564 (9.6%)	12,657 (11.1%)	2065 (9.4%)
*South*	3887 (1.8%)	2730 (2.4%)	264 (1.2%)
*Midwest*	28,706 (13.4%)	15,827 (13.8%)	2250 (10.3%)
*West*	160,356 (75.1%)	83,181 (72.7%)	17,353 (79.1%)
Payer			
*Private*	19,779 (9.3%)	12,835 (11.2%)	4586 (20.9%)
*Medicaid*	163,450 (76.6%)	65,051 (56.9%)	9308 (42.4%)
*Other Public*	2,539 (1.2%)	4366 (3.8%)	953 (4.3%)
*Uninsured*	27,745 (13.0%)	32,143 (28.1%)	7085 (32.3%)
Federal poverty level			
*>138%*	25,998 (12.2%)	14,122 (12.3%)	3376 (15.4%)
*≤138%*	153,885 (72.1%)	82,200 (71.9%)	14,316 (65.3%)
*Unknown*	33,630 (15.8%)	18,073 (15.8%)	4240 (19.3%)
No. of linkages^‡^			
1	206,067 (96.5%)	67321 (58.8%)	14190 (64.7%)
2	7446 (3.5%)	29,697 (26.0%)	4959 (22.6%)
3	—	11,955 (10.5%)	1675 (7.6%)
4	—	3945 (3.4%)	606 (2.8%)
≥5	—	1477 (1.3%)	502 (2.3%)
Time in study, y			
1	62,734 (29.4%)	27,818 (24.3%)	7617 (34.7%)
2	28,656 (13.4%)	12,919 (11.3%)	2599 (11.9%)
3	26,235 (12.3%)	14,110 (12.3%)	2404 (11.0%)
4	18,788 (8.8%)	10817 (9.5%)	1875 (8.5%)
≥5	77,100 (36.1%)	48,731 (42.6%)	7437 (33.9%)
Visits per year			
<1	10,845 (5.1%)	3345 (2.9%)	1330 (6.1%)
(1, 3)	111,252 (52.1%)	40,153 (35.1%)	12,312 (56.1%)
[3, 5)	52,911 (24.8%)	28,781 (25.2%)	4808 (21.9%)
(5, 10)	33,117 (15.5%)	29,855 (26.1%)	2735 (12.5%)
≥10	5388 (2.5%)	12,261 (10.7%)	747 (3.4%)
Chronic conditions^§^			
0	133,970 (62.7%)	37,998 (33.2%)	7905 (36.0%)
1	49,625 (23.2%)	24,985 (21.8%)	4305 (19.6%)
2	18,454 (8.6%)	17,687 (15.5%)	3294 (15.0%)
(3, 5)	9841 (4.6%)	20,612 (18.0%)	3939 (18.0%)
≥5	1623 (0.8%)	13,113 (11.5%)	2489 (11.3%)
Age at death, if applicable			
<1	16 (<0.1%)	—	—
(1, 5)	13 (<0.1%)	—	—
(5,20)	41 (<0.1%)	—	—
(20,40)	12 (<0.1%)	57 (<0.1%)	7 (<0.1%)
(40,60)	—	100 (0.1%)	46 (0.2%)
≥60	—	15 (<0.1%)	13 (0.1%)

For all children, 30% preferred a non-English language, 14% had private health insurance, 69% had Medicaid coverage, and 63% lived in families earning ≤138% federal poverty level (FPL) (see Supplemental Table 1, Supplemental Digital Content, which describes the characteristics of both linked children and all children with a visit).

Eighty-two percent of children linked to a mother only, 14% linked to a father only, and 4% linked to both a mother and a father. Most families consisted of only 1 linked child (61%). Only 2% of children that linked within a family were designated as foster children (see Table 2).

**Table 2:** Characteristics of family units in the cohort.

	Parent combinations, no. (%)		
	Two parents	Mother only	Father only
Characteristic	N = 5430	N = 111,009	N = 18,187
Total children^∗^			
1	4080 (75.1%)	66,395 (59.8%)	12,210 (67.1%)
2	926 (17.1%)	28,462 (25.6%)	3983 (21.9%)
3	273 (5.0%)	11,249 (10.1%)	1228 (6.8%)
4	101 (1.9%)	3614 (3.3%)	422 (2.3%)
5+	50 (0.9%)	1289 (1.2%)	344 (1.9%)
Foster children^†^			
1	206 (3.8%)	2452 (2.2%)	409 (2.2%)
2	35 (0.6%)	622 (0.6%)	73 (0.4%)
3	1 (<0.1%)	189 (0.2%)	16 (0.1%)
4	1 (<0.1%)	47 (<0.1%)	3 (<0.1%)
5+	1 (<0.1%)	13 (<0.1%)	0 (<0.1%)

## 4 Discussion

We applied our previous EHR algorithm (used in Oregon only) to a national network of CHCs. In our previous work using Oregon EHR data from 2002 to 2010, we were able to link 25% of children to a parent using emergency contacts and the guarantor field and validate these linkages.^[13]^ In this cohort, we were able to link 33% of children to a parent from 2007 to 2018 using the same EHR-based data points. The demographics of children and their parents in our identified cohort are similar to the demographics of those seen in CHCs nationally: CHCs serve 1 in 3 people living in poverty, 1 in 5 uninsured persons, 1 in 5 Medicaid beneficiaries, and 1 in 7 racial and ethnic minorities (note these national numbers do not separate adults and children).^[14]^ Thus, we believe this linkage will provide a useful cohort for future research on the parental factors that impact child health.

Previous studies using large datasets were able to identify some parental factors associated with child health care. For example, maternal lack of health insurance was associated with youth lack of health insurance,^[17]^ Affordable Care Act Medicaid expansion was associated with increased odds of well-child care for families making less than 99% FPL,^[18]^ and parent mental illness was associated with potentially preventable child emergency department use.^[19]^ We will expand on these studies by using this rich cohort of linked parent and child EHR data to longitudinally track measures in real-time and investigate the relationships among parental health status and health care utilization and a child's receipt of recommended services and health. Specifically, we plan to assess the association of parent preventive care on receipt of well-child visits. We also intend to study the impact of other important parental factors, like language concordance, chronic disease diagnoses and treatment, and mental health care on their child's receipt of health care and health. Analyses utilizing this cohort will allow examination of the multi-level factors that impact a child's receipt of recommended health care. Once factors are identified, we can make recommendations to inform health policy and primary care practice for improved child health.

These methods have some limitations and methodological issues. We were only able to include children and parents with an encounter in one of the CHCs in the network. We included parents who were 12 to 55 years older than the child, which could have excluded potential parents. We were unable to detect whether the discrepant sex information for child or parent was due to transgender individuals, thus minimizing our ability to include transgender parents (or children). We excluded other relationships that may act similarly to a parent relationship, for example, a grandparent who is raising their grandchild, or a guardian acting as a child's parent. More work is needed to identify broader family definitions using EHR data. We chose a narrow definition of family to include children, mothers, and fathers only, and these exclusions may have removed some child and caregiver pairings.

## 5 Conclusion

We were able to link 33% of children to a parent in EHR data from a large network of community health centers across the United States. Future analyses utilizing these linkages will allow examination of the multi-level factors that impact a child's receipt of recommended health care and health.

## Author contributions

**Conceptualization:** Heather Angier, John Heintzman.

**Data curation:** Sophia Giebultowicz, Jean O’Malley.

**Formal analysis:** Heather Angier, Sophia Giebultowicz, Jorge Kaufmann, Jean O’Malley.

**Funding acquisition:** Jennifer DeVoe.

**Investigation:** Heather Angier, Jorge Kaufmann, John Heintzman.

**Methodology:** Heather Angier, Sophia Giebultowicz, Jorge Kaufmann, John Heintzman, Jean O’Malley.

**Project administration:** Heather Angier, Laura Moreno.

**Supervision:** Heather Angier, John Heintzman, Jennifer DeVoe.

**Visualization:** Heather Angier, Laura Moreno.

**Writing – original draft:** Heather Angier.

**Writing – review & editing:** John Heintzman, Laura Moreno, Jennifer DeVoe.

## Supplementary Material

Supplemental Digital Content
