# Supplementary material for: Creation of a linked cohort of children and their parents in a large, national electronic health record dataset
Source: Medicine (Baltimore). 2021 Aug 13;100(32):e26950. doi: 10.1097/MD.0000000000026950 (PMC8360479; doi:10.1097/MD.0000000000026950)
Supplement: Supplemental Digital Content [file medi-100-e26950-s001.docx]

| Supplemental Table 1. Characteristics of Linked Children and All Children with a Visit | | |
| --- | --- | --- |
|  | **Group, No. (%)** | |
| **Characteristic** | **Linked Children** | **All Children** |
|  | N=213,513 | N=649,894 |
| **Female** | 106,372 (49.8%) | 333,803 (51.4%) |
| **Age at First Encounter^a^** | median=5 | median=8 |
| **Race/Ethnicity** |  |  |
| *Non-Hispanic white* | 58,468 (27.4%) | 183,121 (29.7%) |
| *Non-Hispanic Black* | 30,135 (14.1%) | 104,287 (16.1%) |
| *Hispanic* | 93,020 (43.6%) | 250,742 (38.6%) |
| *Other* | 31,890 (14.9%) | 101,744 (15.7%) |
| **English Language Preferred** | 136,083 (63.7%) | 454,704 (70.0%) |
| **Region^b^** |  |  |
| *Northeast* | 20,564 (9.6%) | 65,439 (10.1%) |
| *South* | 3,887 (1.8%) | 25,827 (4.0%) |
| *Midwest* | 28,706 (13.4%) | 93,634 (14.4%) |
| *West* | 160,356 (75.1%) | 461,589 (71.0%) |
| **Payer** |  |  |
| *Private* | 19,779 (9.3%) | 87,594 (13.5%) |
| *Medicaid* | 163,450 (76.6%) | 449,886 (69.2%) |
| *Other Public* | 2,539 (1.2%) | 8,703 (1.3%) |
| *Uninsured* | 27,745 (13.0%) | 103,583 (16.0%) |
| **Federal Poverty Level** |  |  |
| *>138%* | 25,998 (12.2%) | 86,407 (13.3%) |
| *<=138%* | 153,885 (72.1%) | 411,173 (63.3%) |
| *Unknown* | 33,630 (15.8%) | 152,293 (23.4%) |

| **Note:** 1,570 individuals have dual-roles in this sample, i.e. they are both a *Child* and a *Parent* in separate linkages. Payer and Federal Poverty Level designations are from patient's first encounter or first encounter where data was available. |
| --- |
| ^a^Age range at first study encounter: Children (0-19), Mothers (2-73), Fathers (6-75). Based on all available encounters, where mothers and fathers may have been seen prior to the birth of their first-born child. Only mothers and fathers that were at least 12 years older than their linked child(s) met inclusion criteria. For example, a patient who was age 2 at a 2007 encounter and became a parent in 2017 is included in these data. |
| ^b^States include: Northeast (Massachusetts); South (Florida, Georgia, North Carolina, Texas); Midwest (Indiana, Minnesota, Ohio, Wisconsin); West (Alaska, California, Montana, New Mexico, Nevada, Oregon, Utah, Washington) |
| ^c^The number of parents that children are linked to, or the number of children that mothers and fathers are linked to. |
| ^d^The number of chronic conditions listed in the patient's problem list that were unresolved as of study end. |
